# Inhibition of the Notch Pathway Alleviates Nab-Paclitaxel-Induced Peripheral Neuropathic Pain in Rats by Suppressing HMGB1/Caveolin-1 Signaling in the Spinal Cord

**DOI:** 10.1155/mi/4638804

**Published:** 2025-04-18

**Authors:** Xing Wei, Yaqing Zhou, Li Ma, Weimiao Li, Changyou Shan, Shuqun Zhang, Yonglin Zhao

**Affiliations:** ^1^Department of Gynaecology and Obstetrics, The Second Affiliated Hospital of Xi'an Jiaotong University, Xi'an, China; ^2^Department of Oncology, The Second Affiliated Hospital of Xi'an Jiaotong University, Xi'an, China

**Keywords:** nab-paclitaxel, neuropathic pain, Notch pathway

## Abstract

**Background:** Paclitaxel (PTX) is widely used in the clinical treatment of cancer, and peripheral neuropathy is a common adverse side effect of PTX. Diverse mechanisms contribute to the development and maintenance of PTX-induced peripheral neuropathy. However, the role of the spinal Notch pathway in PTX-induced peripheral neuropathy is not completely understood.

**Methods:** A Sprague–Dawley male rat model of PTX-induced peripheral neuropathy was established by nab-PTX. A total 120 rats were randomly divided into a control group (*n* = 36), PTX d8 group (*n* = 6), PTX d15 group (PTX group, *n* = 36), PTX d21 group (*n* = 6), and PTX+N-[N-(3,5-difluorophenacetyl)-L-alanyl]-S-phenylglycine t-butyl ester (DAPT) (Notch pathway inhibitor) group (*n* = 36). The expression of Notch downstream signaling molecules, including NICD, JAG1, and Hes1 was examined in the control group, PTX d8 group, PTX d15 group, and PTX d21 group. The effects of the DAPT on behavioral assays, apoptosis, neuronal and axonal injury, glial responses, and vascular permeability were detected. The monolayer of mouse brain microvascular endothelial cells was used to simulate vascular permeability in vitro. Cells were divided into the following groups: control group, nab-PTX group, PTX+DAPT group, PTX+DAPT+recombinant mouse high mobility group Box 1 (rmHMGB1) group, and PTX+rmHMGB1+methyl-*β*-cyclodextrin (M*β*CD) group. The underlying mechanisms were explored by examining the expression and translocation of the HMGB1/caveolin-1 signaling pathways, inflammatory cytokines, and oxidative stress in vivo and in vitro.

**Results:** The levels of Notch downstream signaling molecules were elevated and peaked at d15 after nab-PTX treatment. The mechanical and thermal pain thresholds of rats were decreased with nab-PTX treatment, accompanied by enhanced apoptosis, neuronal and axonal injury, glial responses, and vascular permeability. DAPT could restore the mechanical and thermal thresholds and decrease apoptosis, neuronal and axonal injury, and glial responses induced by nab-PTX. DAPT also protected vascular permeability by increasing the expression of tight junction proteins in vivo. RmHMGB1 could abrogate the protective effect of DAPT on vascular permeability, while the inhibitor of caveolin-1, M*β*CD, could further abrogate the effect of rmHMGB1 in vitro. DAPT relieved nab-PTX-induced peripheral neuropathy by inhibiting the activation of the HMGB1/caveolin-1 signaling pathways and decreasing the levels of inflammatory cytokines and oxidative stress in vivo and in vitro.

**Conclusion:** Taken together, the results of this study demonstrated that the Notch pathway may serve as a potential target for PTX-induced peripheral neuropathy intervention.

## 1. Introduction

Paclitaxel (PTX) is a frontline chemotherapeutic reagent for treating solid tumors, such as breast cancer and ovarian cancer. Stabilizing the assembly of intracellular microtubules is one of the mechanisms of the anticancer effect of PTX. Chemotherapy-induced peripheral neuropathy (CIPN) is one of the most common side effects of PTX and occurs in a dose-dependent manner. CIPN usually results in symptoms including burning pain, tingling, numbness, and allodynia in the hands and feet, which significantly affect the quality of life of patients [1]. Compared to standard PTX (Taxol), nab-PTX exhibits a higher administration dose of PTX (175–210 to 260 mg/m^2^), which means a more serious peripheral neuropathy [2]. Treatment options including anti-inflammatory drugs, opioids, and antidepressants usually have limited therapeutic effects. PTX-induced neuropathic pain may be mediated by neuroimmune interactions, mitochondrial dysfunction, and functional upregulation of cation channels in the spinal cord and dorsal root ganglion (DRG). Most research about CIPN focused on pathological changes of DRG and few concentrate on molecular mechanisms in the spinal cord. It has been found that immune responses induced by immune cells and inflammatory factors could contribute to pain hypersensitivity and be involved in the pathogenesis of neuropathic pain in the central nervous system [3]. Microglia are resident immune cells of the central nervous system. The microglia-mediated release of inflammatory mediators is one of the pathophysiological basis for the occurrence of neuropathic pain [4]. For example, dickkopf 3 could ameliorate neuropathic pain via inhibiting ASK-1/JNK/p-38-mediated microglia polarization and neuroinflammation in the spinal cord [5]. In addition, activation of microglia STING signaling pathway could promote the expression of proinflammatory cytokines through TBK1/NF-*κ*B pathway in the spinal cord dorsal horn of spared nerve injury model [6]. Minocycline, known as microglia inhibitor, exerts analgesic effects through various mechanisms and could be a promising therapeutic strategy for chronic pain [7]. However, the mechanisms underlying the development of PTX-induced neuropathic pain are still unknown in the spinal cord.

The Notch signaling pathway has been highly conserved throughout evolution. Many physiological and pathological processes are involved in the Notch pathway, such as neurodegenerative disease, immunological processes, tumor formation, and brain/spinal cord injury [8]. Notch is a transmembrane signaling protein and can be activated by ligands such as Delta and Jagged binding to Notch receptors. After activation, Notch 1 undergoes cleavage close to or within its transmembrane domain by PS1/*γ*-secretase to release the notch intracellular domain (NICD) into the cytoplasm. NICD subsequently translocates to the nucleus, where it regulates transcription. Hairy and enhancer of split 1 (Hes1) is one of the Notch 1 downstream target genes. The Hes1 protein is translated into the cytoplasm and then activates pro-neuronal genes by localizing in the nucleus [9]. A previous study found that the Notch signaling participates in the induction and maintenance of neuropathic pain in the sciatic nerve and spinal cord after spared nerve injury [10]. Notch signaling activation has also been found to contribute to PTX-induced peripheral neuropathy via A1 astrocyte activation [11]. The specific mechanism of the Notch signaling pathway in nab-PTX-induced peripheral neuropathy needs to be further studied.

In this study, we investigated the dynamic expression of the Notch pathway-related proteins after nab-PTX administration. We also assessed the effects of an inhibitor of the Notch pathway on behavioral assays, apoptosis, glial activation, axonal injury, microtubule structure, vascular permeability, inflammatory cytokines, and oxidative stress in the spinal cord in vivo and in the monolayer of endothelial cells in vitro. Our results showed that the expression of the Notch pathway-related proteins was elevated after nab-PTX administration. The protective effect of an inhibitor of the Notch pathway was associated with decreases in the expression and translocation of high mobility group Box 1 (HMGB1), cavolin-1, and inflammatory factors in vivo and in vitro, suggesting that the Notch pathway may be a potential target in nab-PTX-induced peripheral neuropathy treatment.

## 2. Materials and Methods

### 2.1. Experimental Animals and Ethics

All animal experiments were conducted in accordance with ARRIVE guidelines and approved by the Biomedical Ethics Committee of Health Science Center of Xi'an Jiaotong University (No: XJTUAE2023-2321). All efforts were made to minimize discomfort and the number of animals used. A total of 120 male SD rats (250–300 g, 8–10 weeks old) were provided by the Experimental Animal Center of Xi'an Jiaotong University [license no. SCXK (Shaanxi) 2006-001]. Rats were maintained in groups of four per cage under a 12-hour light/dark cycle (07:00 hr on, 19:00 hr off) at a temperature of 25°C with food and water available ad libitum. Rats were adapted to the environment at least 7 days before the experiments.

### 2.2. Establishment of the Nab-PTX-Induced Peripheral Neuropathy Model

To induce neuropathic pain, PTX (Taxol) was injected intraperitoneally on four alternate days (2 mg/kg, with a final cumulative dose of 8 mg/kg) in previous studies [12, 13]. In this study, nab-PTX (Abraxane) was used to simulate a nab-PTX-induced neuropathic pain model. Nab-PTX was obtained from SCPC Pharmaceutical Group Limited Company and dissolved in saline. Since the clinical dose of nab-PTX is 23.8% higher than that of standard PTX [nab-PTX: 260 mg/m^2^ (body surface area) and PTX: 210 mg/m^2^ (body surface area)], the doses of nab-PTX were chosen to be 9.9 mg/kg (the 23.8% higher dose) [14]. Therefore, nab-PTX was injected intraperitoneally on four alternate days (2.47 mg/kg on days 1, 3, 5, and 7 with a final cumulative dose of 9.9 mg/kg) [15]. Control rats were treated with the same dose of saline. No spontaneous abnormal behaviors were observed during or after control or nab-PTX treatment.

### 2.3. Groups and Drug Administration

**Figure 1:**
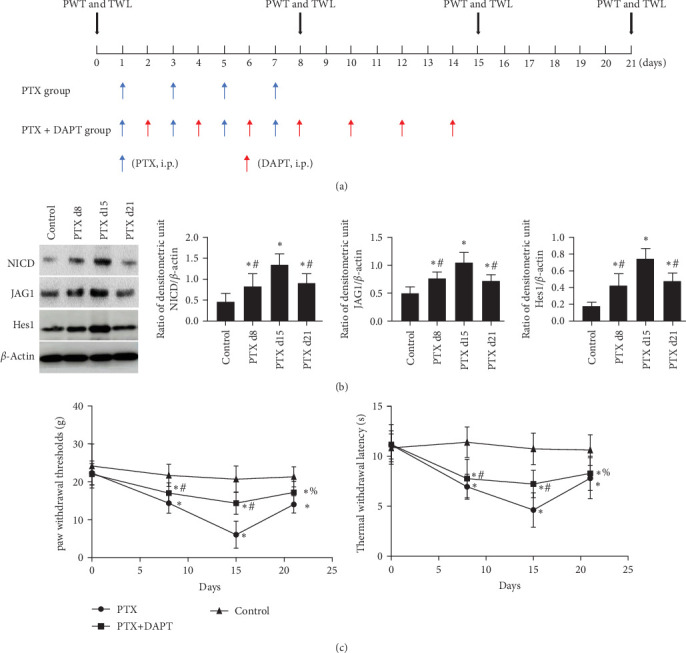
(A) Schematic representation of the experimental procedure. Abbreviations: PTX, nab-paclitaxel; DAPT, N-[N- (3,5-difluorophenacetyl)-L-alanyl]-S-phenylglycine t-butyl ester; i.p., intraperitoneally; PWT, paw withdrawal mechanical threshold; TWL, thermal withdrawal latency. (B) Dynamic expression of proteins related to the Notch signaling pathway after nab-PTX administration. Western blotting analysis was performed to measure the dynamic expression of NICD, JAG1, and Hes1 at d8, d15, and d21 after the first nab-PTX administration. The bar graphs show the expression results for proteins related to the Notch signaling pathway. The expression of *β*-actin was used as an internal control. Values are presented as the mean ± SD (*n* = 6; *⁣*^*∗*^*p* < 0.05 compared to the control group, ^#^*p* < 0.05 compared to the PTX d15 group). (C) DAPT alleviated nab-PTX-induced mechanical and heat hypersensitivity. Paw withdrawal mechanical threshold and TWL of the three groups (*n* = 6) at d0, d8, d15 and d21. *⁣*^*∗*^*p*  < 0.05 compared to the control group. ^#^*p*  < 0.05 compared to the PTX group. ^%^*p*  > 0.05 compared to the PTX group.

Sample size estimation was calculated by a statistical power analysis. A one-way ANOVA was used with the assumption that *α* = 0.05 and power = 0.80. The suggested sample size of each group was 36 (six rats for each experimental project including pathology, TEM, western blot, Evans blue (EB) detection, spinal cord water content, ELISA, and oxidative stress detection). By using a randomized digital table, the total 120 rats (sample size was decided according to a previous study) was randomized into the following groups [15]:

(a) Thirty-six rats were placed in the control group; (b) 36 rats were placed in the PTX group (PTX d15 group), which were treated with nab-PTX and executed on day 15 after the first nab-PTX treatment; (c) six rats were placed in the PTX d8 group, which were treated with nab-PTX and executed on day 8 after the first nab-PTX treatment; (d) six rats were placed in the PTX d21 group, which were treated with nab-PTX and executed on day 21 after the first nab-PTX treatment; and (e) 36 rats were placed in the PTX+N-[N- (3,5-difluorophenacetyl)-L-alanyl]-S-phenylglycine t-butyl ester (DAPT) group, which were treated with nab-PTX and DAPT and executed on day 15 after the first nab-PTX treatment. The *γ*-secretase blocker DAPT was acknowledged as a Notch antagonist. In this group, DAPT solution (1 mg/kg per rat), which was prepared by dissolving DAPT powder (Monmouth Junction, NJ, USA) in 0.01 M phosphate-buffered saline (PBS) containing 5% dimethyl sulfoxide was intraperitoneally injected on seven alternate days (days 2, 4, 6, 8, 10, 12, and 14, Figure 1A) using a microinjection syringe [16–18]. All 120 rats were killed on the scheduled time point after the test of mechanical hyperpathia and heat hypersensitivity. Mechanical hyperpathia and heat hypersensitivity were tested on 0, 8, 15, and 21 days after the first treatment of nab-PTX, respectively. Rats were killed by intraperitoneally injecting 150 mg/kg pentobarbital sodium. The health and behavior of all rats were monitored every day from the beginning to the end of the experiment. The operator ensured that all the rats had no signs of life (no fluctuation in the chest, white eyelids, no visual response, etc.). None of the rats were found died unexpectedly. The order of treatments and measurements is randomized. Apart from the conductor, none was aware of the group allocation at the different stages of the experiment. The duration of the experiment was 10 months.

### 2.4. Behavioral Assays

The paw withdrawal threshold (PWT) and thermal withdrawal latency (TWL) were tested at 0, 8, 15, and 21 days after the first nab-PTX treatment at 1 day (Figure 1). A double-blind match was performed before the tests. Mechanical allodynia was assessed, as previously described [19, 20]. PWT was measured by calibrated von Frey filaments (Stoelting, WoodDale, USA, ranging from 2 to 26 × g bending force). Rats were placed in individual mesh cages and allowed to acclimate for 15 min before measuring the PWT. Von Frey filaments were applied to the ipsilateral midplantar surface perpendicularly, which showed sufficient force to bend against the hind paws for 6–8 s. Every rat received 10 stimulations by a single filament before being pricked by the next larger filament. The interval time between contiguous stimuli was several seconds. Sharp paw withdrawal or paw licking within 5 s was considered a positive response.

TWL of the hind paws was measured using a plantar tester (type 7370; UgoBasile, Varese, Italy), as previously described [21]. Briefly, rats were placed in separate transparent cages, with the bottom being a glass plate. The heating center of an infrared radiant heat stimulus generator (Ugo Basile, Comerio, Italy) was directed at the plantar surface of the hind paw of rats for continuous radiation. The thermal intensity remained unaltered throughout the experiment. The reaction time, defined as the time (s) between the delivery of the thermal stimulus and the withdrawal of the paw, was recorded. Every rat received four tests, with an interval of 5 min between tests, and the first measurement per side was discarded due to it being anomalously long. The average of the test results was used for TWL. There was no difference between the left and right paws.

### 2.5. Hematoxylin–Eosin Staining

Each group of rats was deeply anesthetized with sodium pentobarbital (100 mg/kg) and then intracardially perfused with saline (0.9%), followed by 200 mL of 4% buffered paraformaldehyde. Spinal cord tissue samples (L4–L6) were removed, postfixed, dehydrated, embedded, and sliced. Then, hematoxylin-eosin (H&E) staining was performed. Tissue sections were stained with hematoxylin for 2.5 min and eosin for 16 s, followed by dehydration, hyalinization, and fixation. Finally, H&E staining images were taken by a light microscope (Olympus, Tokyo, Japan) at 40× magnification.

### 2.6. Western Blotting

L4–L6 spinal cords of rats in each group were harvested at corresponding time points. Total protein was isolated using ice-cold RIPA buffer. To detect the nuclear and cytosolic expression of HMGB1, proteins were extracted as directed by the manufacturer using the NE-PER cytoplasmic and nuclear protein extraction kit (Thermo Scientific Pierce, Rockford, IL, USA). Samples were incubated on ice for 30 min, and the supernatants were assembled by centrifugation at 12000 rpm at 4°C. Protein concentrations were determined using the BCA Protein Assay Kit (Thermo Fisher Scientific, MA, USA), according to the manufacturer's instructions. Thirty micrograms of total cell lysate was separated using sodium dodecyl sulfate‒polyacrylamide gel electrophoresis and transferred to polyvinylidene difluoride membranes. Then, the blots were blocked with 5% milk for 1 h at room temperature and probed overnight with the appropriate primary antibodies for mouse monoclonal *β*-actin antibody (1:1000, Cell Signaling Technology, Danvers, MA, USA), rabbit monoclonal cleaved Notch1 (NICD) antibody (1:1000; Cell Signaling Technology, Danvers, MA, USA), Hes1 (1:1000; Abcam, Cambridge, MA, USA), rabbit polyclonal Jagged1 (JAG1) antibody (1:500, Cell Signaling Technology, Danvers, MA, USA), rabbit monoclonal claudin-5 antibody (1:1000, Abcam, Cambridge, MA, USA), rabbit monoclonal PARP1 antibody (1:2000, Sigma‒Aldrich, St. Louis, MO, USA), and rabbit monoclonal occludin-1 antibody (1:1000, Abcam, Cambridge, MA, USA) at 4°C overnight. The membranes were then incubated with horseradish peroxidase (HRP)-conjugated secondary antibody for 1 h. ECL Plus reagents were used for the detection of western blots according to the manufacturer's instructions. Quantification of western blot bands was performed using ImageJ software.

### 2.7. Terminal Deoxynucleotidyl Transferase-Mediated dUTP Biotin Nick End Labeling Assay

A DeadEnd Fluorometric transferase-mediated dUTP biotin Nick end labeling (TUNEL) System (Promega, Madison, Wisconsin, USA) was used to confirm apoptosis according to the manufacturer's instructions. Briefly, L4–L6 spinal cords were fixed and embedded in paraffin, and spinal cord sections of 5 µm thickness were cut using a cryostat. Then, the spinal cord tissue sections were deparaffinized by washing in 100% xylene and a descending ethanol series. Proteinase K was added and incubated for 10 min at room temperature. This was followed by three rinses with PBS and incubation with DAPI for 10 min to stain the nuclei. Images were then taken with a fluorescence microscope. Green TUNEL spots located in the blue-stained nuclei are defined as apoptotic cells. The average total number of cells and apoptotic cells in six sections were counted.

### 2.8. Immunohistochemistry

The paraffin-embedded L4–L6 spinal cords of rats in each group were cut at a thickness of 5 μm. Paraffin-embedded sections were deparaffinized with xylene, rehydrated in graded ethanol solutions and washed in water. Sections were then boiled in a microwave with citrate buffer (pH 6.0) for 30 min to retrieve antigens. Endogenous peroxidase and nonspecific binding were blocked by incubating the slides in 3% hydrogen peroxide and 3% bovine serum albumin for 30 min. The sections were then incubated at 4°C overnight with the respective primary antibodies diluted in normal antibody diluent: mouse monoclonal glial fibrillary acidic protein (GFAP) (1:400, Cell Signaling Technology, Danvers, MA, USA), rabbit polyclonal Iba-1 (1:300, Wako, Tokyo, Japan), rabbit monoclonal neurofilament (NF) light chain (NF-L, 1:100, Cell Signaling Technology, Danvers, MA, USA), mouse monoclonal NF heavy chain (NF-H, 1:100, Cell Signaling Technology, Danvers, MA, USA), mouse monoclonal NF medium chain (NF-M, 1:100, Cell Signaling Technology, Danvers, MA, USA), and rabbit polyclonal HMGB1 (1:400, Cell Signaling Technology, Danvers, MA, USA). After primary antibody incubation, the sections were washed and incubated for 1 h in HRP-conjugated secondary antibodies (1:500). 3,3′-Diaminobenzidine solution was used to detect HRP activity for 10 min. Finally, the sections were dehydrated in ascending alcohol concentrations and xylene and covered with coverslips. The number of positive cells was calculated in six random microscopic fields of each section (three sections per animal) by ImageJ software. The result of immunohistochemical staining was recorded as a score based on the number of positive cells and staining intensity. Immunohistochemical scores (IHSs) were determined by multiplying the quantity and staining intensity scores as follows: (1) the quantity of positive cells was graded from 0 to 4, with no staining, 0; 1%–10% of cells stained, 1; 11%–50%, 2; 51%–80%, 3; and 81%–100%, 4; and (2) staining intensity was graded from 0 to 3, with 0 = negative, 1 = weak, 2 = moderate, and 3 = strong. Theoretically, the scores could range from 0 to 12 [22].

### 2.9. Immunofluorescence Staining

Briefly, paraffin-embedded sections were deparaffinized with xylene, rehydrated in graded ethanol solutions and washed in water. Sections were inactivated of endogenous peroxidase, blocked with 10% donkey serum, and probed with anti-caveolin-1 (1:400, Cell Signaling Technology, Danvers, MA, USA) and anti-ZO-1 (1:200, Abcam, Cambridge, MA, USA) antibodies overnight at 4°C. The sections were washed and incubated with fluorochrome-conjugated secondary antibodies. The samples were then counterstained with DAPI and imaged using a fluorescence microscope.

### 2.10. Transmission Electron Microscopy

L4–L6 spinal cords of rats in each group were obtained on Day 15 after deep anesthesia with sodium pentobarbital (100 mg/kg). Neuronal ultrastructural changes in the L4–L6 spinal cords of rats were evaluated by transmission electron microscopy. L4–L6 spinal cords were cut into 1 mm^3^ sections and immediately fixed in glutaraldehyde (2.5%) overnight. Then, the tissue was washed, fixed, dehydrated and soaked in linoleate at 60°C overnight. The tissue was cut into semithin sections. Semithin sections were cut into thin sections (50–70 nm) after methylene blue staining. Then, the microtubules in spinal cord neurons were observed by transmission electron microscopy (H-7650, Hitachi, Tokyo, Japan).

### 2.11. Enzyme-Linked Immunosorbent Assay

L4–L6 spinal cords of rats in each group were rapidly isolated on Day 15 after deep anesthesia with sodium pentobarbital. Spinal cords were homogenized with ethylene-diamine tetraacetic acid-free complete protease inhibitor cocktail tablets using 50 *μ*L/10 mg tissue. After 4000 *r*/min centrifugation for 15 min at 4°C, the total protein concentrations of the supernatant were measured using a BCA Protein Assay Kit (Thermo Fisher Scientific, Carlsbad, CA, USA). Concentrations of proinflammatory factors (interleukin [IL-1*β*], tumor necrosis factor [TNF-*α*]) and anti-inflammatory factors (IL-4, IL-10) were measured by rat-specific ELISA kits (R&D Systems, Minneapolis, MN, USA) according to the manufacturer's instructions. Each sample was run in duplicate, and data (pg protein) were normalized to mg of total protein.

### 2.12. Vascular Permeability Assay

EB staining was performed to assess the vascular permeability of the spinal cord. EB (Sigma-Aldrich, St. Louis, MO, USA) was intravenously injected with 4 mL/kg of 2% EB via the tail vein 1 h. Approximately 1 h after circulation, the rat was transcardially perfused with saline, and the L4−L6 spinal cord was quickly removed. The EB content was determined, as previously described [23]. The spinal cord was immediately weighed, homogenized, and then incubated in formamide (24 h, 55°C). The homogenate supernatant was diluted and measured at 620 nm. The EB concentrations were normalized based on the results from spinal cord samples from the control groups.

### 2.13. Evaluation of Edema

To evaluate the water content of the spinal cord, the wet‒dry method was used. Briefly, the spinal cord was collected and weighed as the wet weight. The spinal cords were then fully dried in an oven at a constant temperature of 100°C for 72 h. After that, the spinal cords were weighed several times to obtain a stable dry weight. The percentage of spinal cord water content was calculated as (wet weight − dry weight)/wet weight × 100%.

### 2.14. Cell Culture and Treatments

The bEnd.3 cell line was immortalized from mouse brain microvascular endothelial cells and obtained from the American Type Culture Collection. Cells were grown in DMEM supplemented with 10% fetal bovine serum and 1% penicillin/streptomycin. Cells were maintained in a humidified incubator at 37°C with 5% CO_2_ and 95% air. Cells were seeded onto Transwells with 0.4 μm pore polyester membrane inserts in 24-well plates at a density of 1 × 10^5^ to 1 × 10^6^ cells/cm^2^.

Cells were divided into the following groups: control group, nab-PTX group, PTX+DAPT group, PTX+DAPT+recombinant mouse high mobility group Box 1 (rmHMGB1) group, and PTX+rmHMGB1+methyl-*β*-cyclodextrin (M*β*CD) group. In the nab-PTX (PTX) groups, bEnd.3 cells were cultured on the inner surface of collagen-coated Transwell inserts and treated with nab-PTX (24.7 μmol/L) for 5 h [24, 25]. Since the clinical dose of nab-PTX is 23.8% higher than that of standard PTX, the doses of nab-PTX were chosen to be 24.7 mg/kg (20 μmol/L). In the PTX+DAPT groups, DAPT was added and incubated for 24 h at a final concentration of 20 μmol/L [26]. In the PTX+DAPT+rmHMGB1 group, mouse recombinant HMGB1 (mrHMGB1, Abcam, Cambridge, MA, USA) was added and incubated for 8 h at a final concentration of 1 μg/mL [27, 28]. In the PTX+rmHMGB1+M*β*CD group, after forming a confluent monolayer, bEnd.3 cells were then incubated with M*β*CD (5 mM, Sigma‒Aldrich, St Louis, MO, USA) for 1 h before nab-PTX and rmHMGB1 treatment [29]. M*β*CD is a water-soluble heptasaccharide that acts as a cholesterol scavenger by binding to cholesterol through a hydrophobic core and has been considered an inhibitor of caveolin-1.

### 2.15. Transendothelial Electrical Resistance Measurement

Transendothelial electrical resistance (TEER) was measured using a Millicell-ERS instrument (Millipore, Billerica, MA, USA). The resistance of blank filters was subtracted from that of cell-coated filters before the final resistance values were calculated. The integrity of the endothelial monolayer in Transwell inserts was assessed for 5 days. bEnd.3 cells were replenished with fresh medium every 2 days. TEER was reported in μ*Ω*/cm^2^.

### 2.16. Horseradish Peroxidase Flux

HRP flux experiments were used to measure endothelial cell permeability in vitro. One mL culture medium containing 10 mg/mL HRP (0.5 mM, SigmaAldrich, St. Louis, MO, USA) was added to the upper system, and 2 mL of culture medium was added to the well. The mixture was incubated at 37°C for 1 h. Then, the culture medium was collected from the lower compartment. The amount of HRP in the lower chamber was quantified, as described previously. HRP flux was assumed to be in nanograms per milliliter.

### 2.17. Measurement of the Activities of Catalase, Superoxide Dismutase, Malondialdehyde, and Glutathione Peroxidase

All spinal cord samples were homogenized and centrifuged at 4,000 × rpm for 15 min at 4°C and then analyzed to detect the activities of malondialdehyde (MDA), catalase (CAT), superoxide dismutase (SOD), and glutathione peroxidase (GSH) following the manufacturer's instructions (Nanjing Jiancheng Bioengineering Institute, Nanjing, China).

### 2.18. Statistical Analysis

SPSS 18.0 (SPSS, Chicago, IL, USA) was used for statistical analysis. All data are presented as the mean ± SD. Behavioral data including PWT and TWL were analyzed using two-way repeated measures ANOVA followed by Tukey post-hoc test. Biochemical measurements, including western blot, immunofluorescence density, immumohistochemical staining, ELISA, and so on, were compared using one-way ANOVA followed by Tukey post-hoc test. The differences are given in the bar graphs. A *p* value less than 0.05 was considered statistically significant.

## 3. Results

1. Dynamic expression of proteins related to the Notch signaling pathway and effects of the Notch pathway inhibitor DAPT on nab-PTX-induced neuropathic pain.

Western blotting analysis was performed to examine the expression of proteins related to the Notch signaling pathway, including NICD, JAG1, and Hes1, at different time points. The results showed that compared to the control group, the expression of these proteins was significantly upregulated in the spinal cord on d8, d15, and d21 after the first nab-PTX administration (*p* all  < 0.001). Compared to the PTX d15 group, the level of these proteins was significantly lower in the PTX d8 group and PTX d21 group (*p* all  < 0.001) (Figure 1B).

The rats received nab-PTX on days 1, 3, 5, and 7. Mechanical allodynia and heat hypersensitivity were tested at 0 days before nab-PTX administration and at 8 days (d8), 15 days (d15), and 21 days (d21) after the first nab-PTX administration. Compared to the control group, the PWT and TWL were significantly decreased in the PTX group at d8, d15, and d21 (*p* all  < 0.001). Compared to the PTX group at d8 and d15, PWT and TWT were significantly increased in the PTX+DAPT group (*p* all  < 0.001). There was no significant difference in PWT and TWT between the PTX group and PTX+DAPT group at d21 (*p* both  > 0.05) (Figure 1C). The results revealed that the Notch pathway inhibitor DAPT could alleviate mechanical and heat hypersensitivity induced by nab-PTX. Considering the tendency of changes in the expression of proteins related to the Notch signaling pathway and in PWT and TWT, the PTX d15 group (PTX group) was used to conduct further studies.

2. Role of the Notch pathway in axonal pathological changes and microtubule histomorphology in the rat spinal cord after nab-PTX administration.

**Figure 2:**
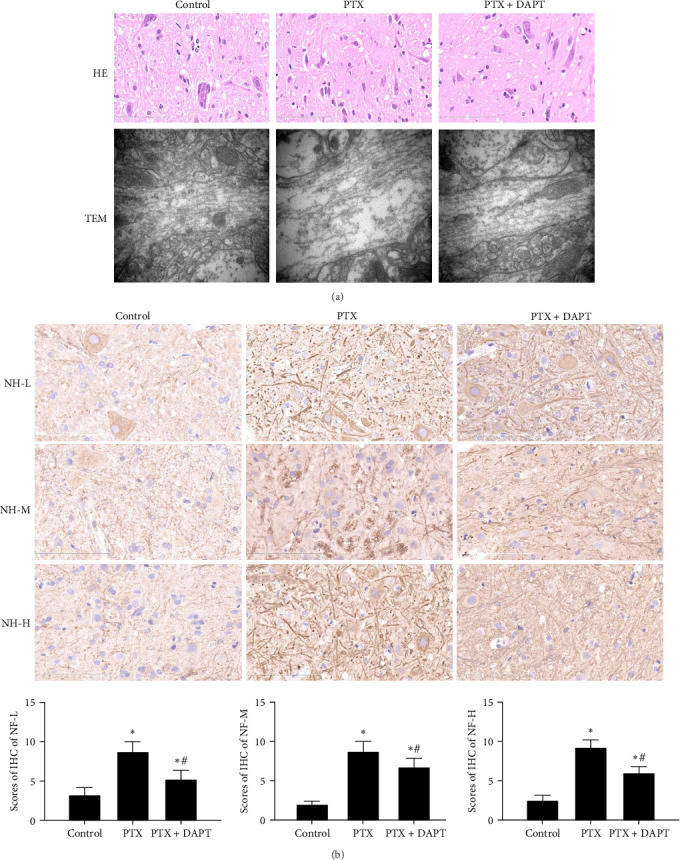
(A) DAPT attenuates pathological changes and protects against ultrastructural alterations in microtubule histomorphology. Pathological changes in all groups were confirmed by H&E staining (scale bars = 100 μm, *n* = 6). Ultrastructural alterations of the longitudinal sections of microtubules after nab-PTX administration were observed by TEM (scale bars = 500 nm, *n* = 6). (B) Protective effect of DAPT in axons after nab-PTX administration. Pathological changes in axons were assessed by NF-L, NF-M, and NF-H immunohistochemistry. Scale bars = 100 μm. Bar graphs show the NF-L, NF-M, and NF-H immunohistochemistry results evaluated by IHS. Values are presented as the mean ± SD (*n* = 6; *⁣*^*∗*^*p* < 0.05 compared to control group, ^#^*p* < 0.05 compared to PTX group).

Histopathological features were examined to evaluate the pathological changes in the rat spinal cord by H&E staining and microtubule histomorphology by TEM. In the H&E-stained sections, no abnormalities were observed in the control group. In the PTX group, H&E staining revealed neuronal pyknosis, torsion, and cell body deformation in the spinal cord of rats. Compared to the PTX group, the pathological changes, including neuronal pyknosis, torsion, and cell body deformation, were reduced in the PTX+DAPT group. The structure of microtubules was found to be consecutive, integrated, and compact in the control group. In the PTX group, the microtubules were fractured and discontinuous and displayed obvious free ends. Compared to the PTX group, the number, integrity, and compactness of microtubules were preserved (Figure 2A).

NF-L, NF-M, and NF-H are considered markers of axonal injury. To investigate the role played by the Notch pathway in axonal injury after nab-PTX administration, the expression of NF-L, NF-M, and NF-H was assessed by immunostaining in the rat spinal cord. NF-L, NF-M, and NF-H were rarely detected in the control group. In contrast, compared to expression in the control group, the expression of NF-L, NF-M, and NF-H was increased in the PTX group (*p* all  < 0.001). Compared to expression in the PTX group, DAPT decreased the expression of NF-L, NF-M, and NF-H in the PTX+DAPT group (*p* all  < 0.001) (Figure 2B).

3. Notch pathway inhibition significantly ameliorated apoptosis and glial response after nab-PTXadministration.

**Figure 3:**
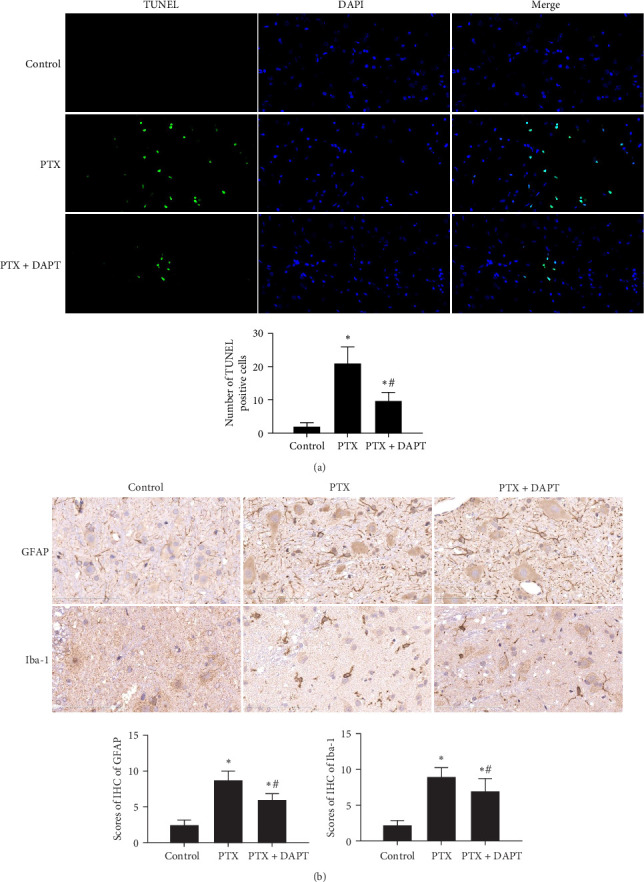
(A) Inhibition of the Notch pathway significantly attenuates apoptosis after nab-PTX administration. Apoptotic cells were detected by TUNEL assay. TUNEL-positive cells were stained green, and the nuclei were stained with DAPI (blue). Bar graphs show the numbers of apoptotic cells that were counted in five random cortical fields (×40 magnification). (B) Effects of DAPT on the expression levels of glial response markers (Iba-1 and GFAP). GFAP and Iba-1 were assessed via immunohistochemistry in the rat spinal cord. Scale bars = 100 μm. The bar graphs show the statistical analysis of the IHS of Iba-1- and GFAP-positive cells in the different experimental groups. All values are presented as the mean ± SD (*n* = 6; *⁣*^*∗*^*p* < 0.05 compared to the control group, ^#^*p* < 0.05 compared to the PTX group).

Apoptosis was detected by TUNEL assay in the spinal cord of rats. Few TUNEL-positive cells were detected in the control group. Compared to the control group, TUNEL-positive cells were evident in the PTX group (*p* < 0.001). Compared to the PTX group, the number of apoptotic cells was significantly decreased in the PTX+DAPT group (*p* < 0.001) (Figure 3A).

Compared to the control group, the IHS of GFAP and Iba-1, assessed by the number and staining intensity of Iba-1-positive microglial cells and GFAP-positive astrocytes, was significantly increased in the PTX group (*p* both  < 0.001). Compared to the PTX group, staining and numbers of Iba-1-positive microglia and GFAP-positive astrocytes were significantly decreased in the PTX+DAPT group (*p* both  < 0.001) (Figure 3B).

4. Inhibition of the Notch pathway protected vascular permeability after nab-PTX administration.

**Figure 4:**
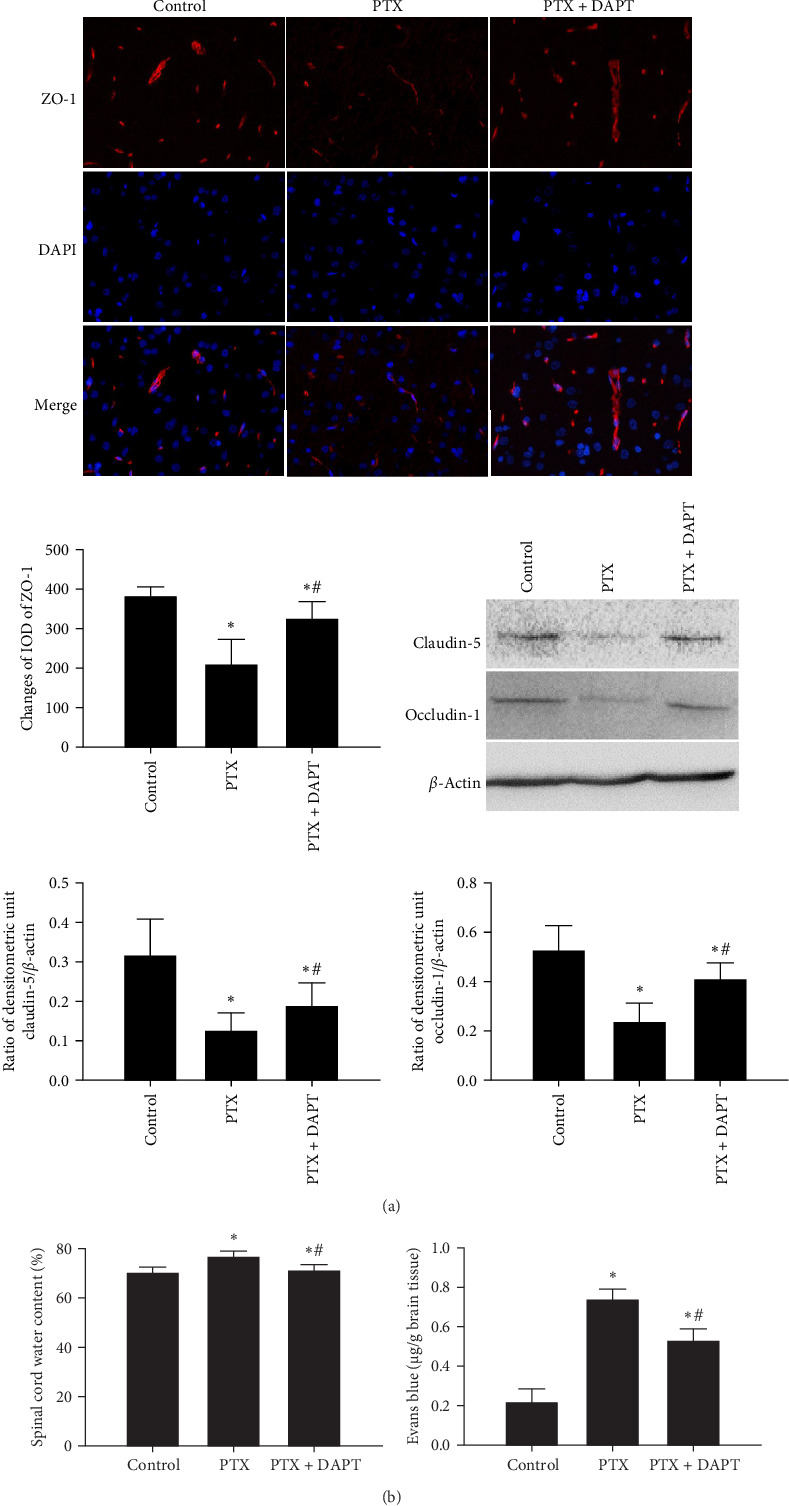
(A) Effects of DAPT on the expression levels of ZO-1, claudin-5, and occludin-1 proteins after nab-PTX administration. ZO-1 was assessed via immunofluorescence in the rat spinal cord. Scale bars = 100 μm. The expression of claudin-5 and occludin-1 in the spinal cord was examined by western blotting. The expression of *β*-actin was used as an internal control. The bar graphs show the statistical results of the expression of ZO-1, claudin-5, and occludin-1. (B) Vascular permeability was assessed by EB diffusion and the water content of the spinal cord. All values are presented as the mean ± SD (*n* = 6; *⁣*^*∗*^*p* < 0.05 compared to the control group, ^#^*p* < 0.05 compared to the PTX group).

Immunofluorescence staining was used to detect the expression of ZO-1, and western blotting analysis was performed to measure the expression levels of claudin-5 and occludin-1. The results showed that the expression of ZO-1, claudin-5, and occludin-1 was decreased following nab-PTX administration compared to that in the control group (*p* all  < 0.001), indicating that vascular integrity was disrupted after nab-PTX administration. DAPT treatment resulted in significantly higher levels of ZO-1, claudin-5, and occludin-1 expression than PTX treatment (*p* all  < 0.001) (Figure 4A).

EB and water content were used to monitor the destruction of vascular integrity and edema of the spinal cord. Little EB diffusion was detected in the control group. Compared to the control group, nab-PTX treatment induced significant brain edema and leakage of EB (*p* both  < 0.001), which were significantly alleviated by DAPT treatment in the PTX+DAPT group (*p* both  < 0.001) (Figure 4B).

5. The neuroprotective effects of DAPT were related to the expression of HMGB1, cavolin-1, and downstream inflammatory cytokines and oxidative stress.

**Figure 5:**
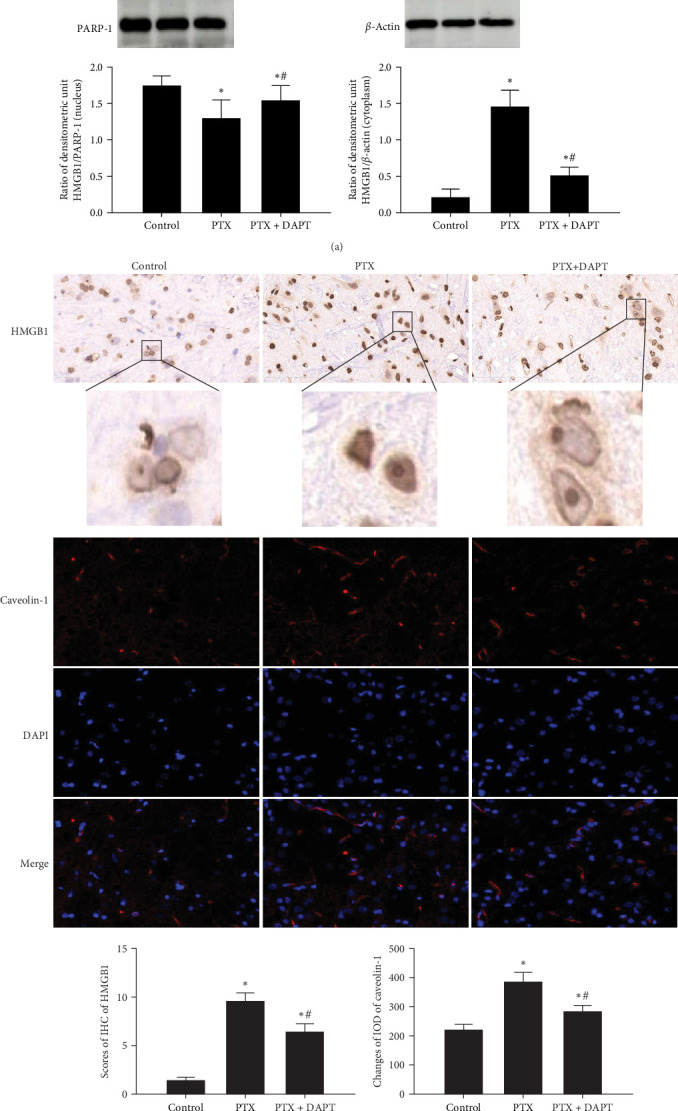
The translocation and expression of HMGB1 and caveolin-1 are involved in nab-PTX-induced peripheral neuropathy. (A) HMGB1 translocation was detected by western blotting in the nucleus and cytoplasm. (B) The expression of HMGB1 and caveolin-1 was determined by immunohistochemistry and immunofluorescence, respectively. Bar graphs show the HMGB1 and caveolin-1 immunoassay results. Immunoreactivity was assessed in five random fields (×40 magnification) via IHS or integral optical density (IOD) values. All values are presented as the mean ± SD (*n* = 6; *⁣*^*∗*^*p* < 0.05 compared to the control group, ^#^*p* < 0.05 compared to the PTX group).

To determine the downstream targets of the Notch pathway after nab-PTX administration, HMGB1 immunoassays, and caveolin-1 immunostaining were performed in the rat spinal cord. Western blotting analysis showed that in the control group, HMGB1 was mainly expressed in the nucleus. Compared to the control group, the expression of HMGB1 was decreased in the nucleus and increased in the cytoplasm in the PTX group (*p* both  < 0.001). Compared to the PTX group, the expression of HMGB1 was increased in the nucleus and decreased in the cytoplasm in the PTX+DAPT group (*p* both  < 0.001) (Figure 5A). The staining showed that compared to the control group, the expression of HMGB1 and caveolin-1 was increased, and cytosolic translocation of HMGB1 was also observed after nab-PTX treatment (*p* both  < 0.001). Compared to the PTX group, the expression of caveolin-1 and HMGB1 was decreased in the PTX+DAPT group (*p* both  < 0.001). The cytosolic translocation of HMGB1 was also decreased (*p* < 0.001) (Figure 5B).

**Figure 6:**
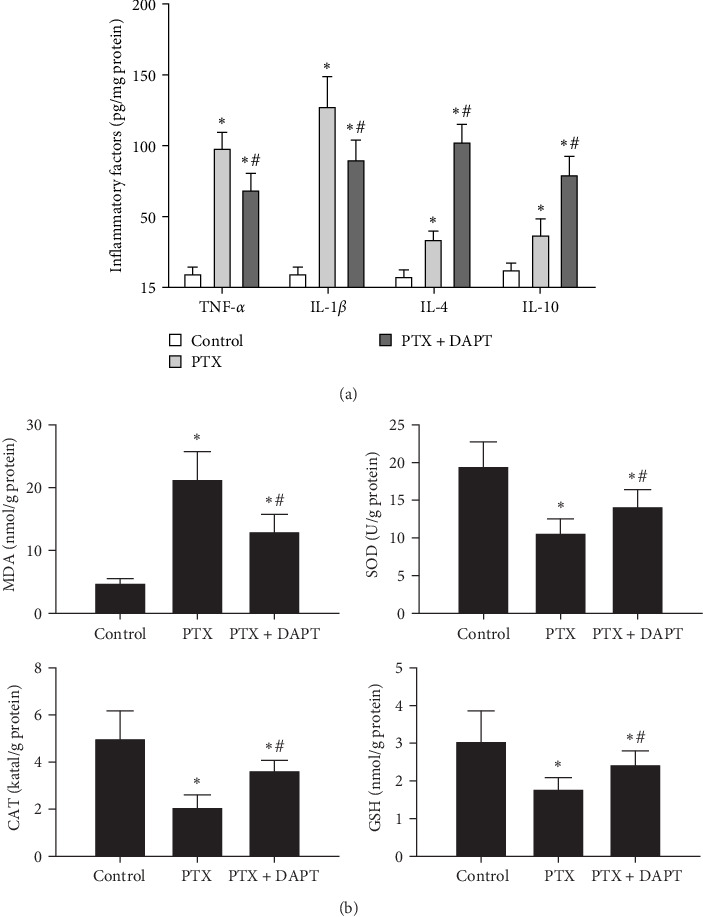
(A) Effects of DAPT on the levels of inflammatory factors, including TNF-*α*, IL-1*β*, IL-4, and IL-10, in the rat spinal cord after nab-PTX-induced peripheral neuropathy were determined by ELISA. (B) The levels and activities of SOD, CAT, MDA, and GSH in the spinal cord represent oxidative stress. Values are presented as the mean ± SD (*n* = 6; *⁣*^*∗*^*p* < 0.05 compared to control group, ^#^*p* < 0.05 compared to PTX group).

The levels of proinflammatory factors, such as TNF-*α* and IL-1*β*, and anti-inflammatory factors, such as IL-4 and IL-10, in the spinal cord of rats after nab-PTX administration were determined by ELISA. The results showed that the levels of inflammatory factors (TNF-*α*, IL-1*β*, IL-4, and IL-10) were increased in the PTX group compared with the control group (*p* all  < 0.001). Compared to the PTX group, the levels of proinflammatory factors were decreased, and the levels of anti-inflammatory factors were further increased in the PTX+DAPT group (*p* all  < 0.001) (Figure 6A). Oxidative stress was also detected through the levels of MDA, SOD, GSH, and CAT. Compared to the control group, the levels of MDA in the PTX group were significantly increased, and the levels of SOD, GSH and CAT were significantly reduced (*p* all  < 0.001). After DAPT treatment, the levels of MDA were significantly reduced, and the levels of SOD, GSH, and CAT were significantly increased compared to those in the PTX group (*p* all  < 0.001) (Figure 6B).

6. The role of Notch signaling in vascular permeability after nab-PTX administration was related to the HMGB1/cavolin-1 pathway.

**Figure 7:**
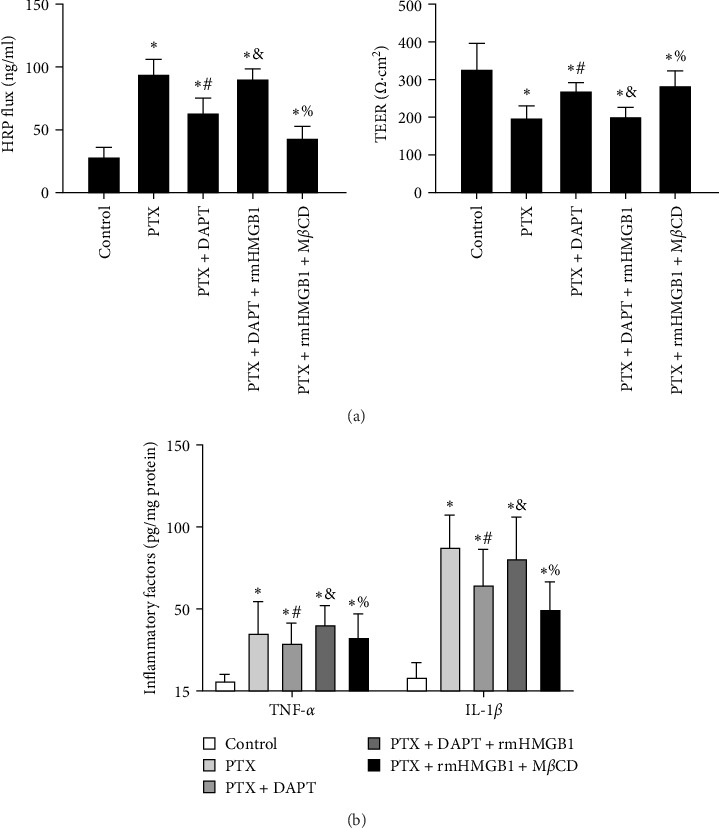
(A) TEER and HRP flux were measured to assess the integrity of the monolayer of endothelial cells in vitro. (B) The levels of TNF-*α* and IL-1*β* in monolayers of endothelial cells after nab-PTX treatment were determined by ELISA in different groups. All values are presented as the mean ± SD (*n* = 6; *⁣*^*∗*^*p* < 0.05 compared to the control group, ^#^*p* < 0.05 compared to the PTX group, ^&^*p* < 0.05 compared to the PTX+DAPT group, ^%^*p* < 0.05 compared to the PTX+DAPT+rmHMGB1 group).

TEER and HRP flux were measured to assess the integrity of the monolayer of endothelial cells in response to different stimuli and treatments. Compared to the control group, the TEER decreased, whereas the HRP flux increased in the PTX group (*p* both  < 0.001). DAPT treatment significantly increased TEER and decreased HRP flux (*p* both  < 0.001). rmHMGB1 is considered an exogenous protein that activates the downstream pathway of HMGB1. Compared to the PTX+ APT group, the TEER decreased, and the HRP flux increased in the PTX+DAPT+rmHMGB1 group (*p* both  < 0.001), indicating that rmHMGB1 treatment abolished the protective effect of DAPT. Compared to the PTX+DAPT+rmHMGB1 group, the TEER increased, and the HRP flux decreased in the PTX+rmHMGB1+M*β*CD group (*p* both  < 0.001), indicating that inhibition of caveolin-1 could protect against the vascular hyperpermeability induced by HMGB1 (Figure 7A).

The levels of the proinflammatory factors TNF-*α* and IL-1*β* in the monolayer of endothelial cells after nab-PTX administration were determined by ELISA. The results showed that the levels of TNF-*α* and IL-1*β* were increased in the PTX group compared with the control group (*p* both  < 0.001). Compared to the PTX group, the levels of TNF-*α* and IL-1*β* were decreased in the PTX+DAPT group (*p* both  < 0.001). Compared to the PTX+DAPT group, the levels of TNF-*α* and IL-1*β* were increased in the PTX+DAPT+rmHMGB1 group (*p* both  < 0.001). Compared to the PTX+DAPT+rmHMGB1 group, the levels of TNF-*α* and IL-1*β* were decreased in the PTX+rmHMGB1+M*β*CD group (*p* both  < 0.001) (Figure 7B).

## 4. Discussion

There are limited treatment options for nab-PTX-induced peripheral neuropathy. Currently, the pathogenesis of nab-PTX-induced peripheral neuropathy is unclear. In this study, we discovered that the expression of the Notch pathway-related proteins was significantly higher after nab-PTX administration. The Notch pathway inhibitor DAPT alleviated nab-PTX-evoked pain, including mechanical allodynia and thermal hypersensitivity, spinal cord apoptosis, glial response, oxidative stress, and microtubule and axonal damage, in a rat PTX-induced peripheral neuropathy model by protecting vascular integrity and decreasing the levels of proinflammatory cytokines in vivo and in vitro via the Notch/HMGB1/caveolin-1 pathway.

In this study, a PTX-induced peripheral neuropathy model was established by nab-PTX administration at a higher dose [12, 13]. The results showed that the PWT and TWL were significantly decreased in the PTX group which demonstrated that the nab-PTX-induced peripheral neuropathy model was successfully established. A previous study also found that both mechanical and cold allodynia could be induced by repeated administration of nab-PTX, and the effects of nab-PTX on pain behaviors tended to be stronger than those of standard PTX at the doses used clinically [14].

Notch signaling has an important role in synaptic plasticity and inflammation in the central nervous system. Previous studies have found that activation of the Notch is correlated with neuropathic pain, including PTX-induced peripheral neuropathy. Notch signaling activation contributes to PTX-induced neuropathic pain via the activation of A1 astrocytes [11]. Pregabalin and lacosamide could ameliorate PTX-induced peripheral neuropathy via inhibition of JAK/STAT signaling pathway and Notch-1 receptor [30]. The activation of the Notch pathway can cause the release of pro-inflammatory factors, synaptic transmission, and calcium inward flow, which play a key role in the development of neuropathic pain. In this study, we innovatively found that the Notch pathway is associated with the destruction of vascular integrity. Inhibition of the Notch pathway could lessen the permeability of vascular by decreasing inflammatory cytokines and oxidative stress and through HMGB1/caveolin-1 signaling, which further defined the relation between activation of the Notch pathway and the development of nab-PTX-induced peripheral neuropathy. JAG1 is an important Notch ligand to promote activation of the Notch pathway. NICD is released from the cell membrane and enters the nucleus to activate transcription of its downstream genes. Previous studies found the protein expression of spinal Jagged1, NICD, and Hes1 was markedly increased in the rat PTX-induced neuropathic pain model, which is consistent with the results of this study. These results further confirmed that the Notch signaling pathway is activated by nab-PTX and participates in the process of nab-PTX-induced peripheral neuropathy.

NF: NF-L, NF-M, and NF-H are axonal structural cytoskeletal components that are important for neuronal electric signal transmission along the axons. Abnormal assembly of neurofilaments is found in several human neurodegenerative diseases, including peripheral neuropathy [31]. Both the mRNA and protein levels of NF-L were significantly higher in the L4–L5 dorsal spinal cord DRG tissues of rats that received spinal nerve ligation. Moreover, spinal neuropathic pain was ameliorated by repressing the NF light polypeptide [32]. In this study, pathological changes in axons were assessed, and the results showed that DAPT attenuated the expression of NF-L, NF-M, and NF-H and protected against ultrastructural alterations in microtubule histomorphology. A previous study found that in a mouse PTX model of CIPN, axonal function was lost. The irreversible sterile alpha and TIR motif containing one inhibitor prevented the loss of intraepidermal nerve fibers induced by PTX and provided partial protection of axonal function [33]. All the results led to an increased recognition of the importance of axon damage in the pathophysiology of nab-PTX-induced peripheral neuropathy.

The cytotoxic agent PTX on the normal microvasculature has been established. PTX significantly inhibited tube formation and proliferation and increased endothelial necrosis in human dermal microvascular endothelial cells [34]. Monolayer brain endothelial bEnd.3 cells were used to evaluate the role of nab-PTX in the disruption of microvasculature [35, 36]. Our previous study found that inhibition of soluble epoxide hydrolase could protect vascular integrity by increasing the expression of tight junction proteins [15]. Claudin-5 is essential to the structural integrity of the blood-spinal cord barrier, and the integrity of the blood–spinal cord barrier could be improved by reducing the degradation of ZO-1 by reversing vasogenic edema and leakage [37]. Occludin is also an essential component of tight junctions, which are involved in controlling the integrity of the blood-spinal cord barrier [38]. In this study, inhibition of the Notch pathway also reversed the decreased expression of ZO-1, claudin-5, and occludin-1 induced by nab-PTX administration, indicating that vascular integrity was protected by DAPT. Similarly, the expression of inflammatory cytokines was significantly increased, the expression of ZO-1 and claudin-4 proteins was decreased, and alveolar-capillary membrane permeability was increased in lung tissue after PTX treatment [39]. These results showed that PTX-induced peripheral neuropathy was related to the disruption of microvasculature. However, several studies found that PTX could promote the accumulation of junctional proteins at the cell cortex and the stabilization of microtubules by rescuing the barrier after cytokine treatment in three-dimensional culture models of the epidermis [40]. These contradictory views seem to be related to different disease models and pathophysiological microenvironments, which need further study.

HMGB1 is a nuclear and cytosolic protein that can act as a transcription factor. HMGB1 was found to be localized primarily in cell nuclei and partially in cytoplasmic compartments in the normal cerebral cortex. The cytosolic HMGB1 level could be significantly increased and nuclear HMGB1 decreased after several CNS diseases including subarachnoid hemorrhage and ischemia, which suggested that the early cytosolic HMGB1 increase was predominantly due to the translocation of nuclear HMGB1. In this study, the nuclear and cytosolic expression of HMGB1 was detected after extraction using the cytoplasmic and nuclear protein extraction kit. The analysis showed that HMGB1 was mainly expressed in the nucleus under physiological conditions. The expression of HMGB1 was decreased in the nucleus and increased in the cytoplasm after nab-PTX treatment. The expression of HMGB1 was increased in the nucleus and decreased in the cytoplasm after DAPT treatment indicated by western and immunohistochemistry. In this study, the expression of HMGB1 was suppressed by DAPT, which means that HMGB1 was one of the downstream targets of Notch signaling. A previous study found that Notch activation was universal in proliferating astrocytes and that the Notch ligand Jagged1 was uniquely upregulated in proliferating microglia [41]. Disruption of Notch signaling by DAPT attenuated lipopolysaccharide-induced inflammatory responses, including HMGB1 [42]. Conversely, DAPT treatment could upregulate the protein expression of HMGB1 and NF-*κ*B in acetaminophen-challenged livers [43]. In HMGB1-treated mouse hearts, Notch1 and Hes1 expression was increased by HMGB1, and the Notch pathway is involved in HMGB1-induced effects on cardiac regeneration [44]. It seems that the regulatory effects of the Notch pathway on HMGB1 are related to different organ damage and diseases. It has been indicated that CIPN could be prevented by neutralization of HMGB1 with monoclonal antibodies [45]. In this study, DAPT treatment decreased the level of proinflammatory factors and increased the level of anti-inflammatory factors, indicating the interaction between the immune system and neuropathic pain. The administration of TNF-*α* and IL-1*β* antagonists has been found to decrease behaviors suggestive of pain and hyperalgesia in rodents following chronic constriction injury [46]. However, whether microglia are activated after PTX treatment is controversial. In several studies, spinal astrocytes and microglia were activated in PTX-treated rats [12, 47]. However, there was no microglial hypertrophy or increased Iba-1 staining in animals treated with PTX, vincristine, or oxaliplatin [48]. The reasons for this discrepancy may be related to different diseases, rodent models, and detection means. DAPT could inhibit the activation of astrocytes. Previous studies found activated microglia induce A1 astrocytes by secreting Il-1*α* and TNF, and the activation of microglia was also suppressed in this study [49]. Pharmacologically blocking Notch1 signaling with DAPT can induce in situ astrocyte-to-neuron conversion after spinal cord injury [50]. The downstream molecules of Notch signaling were colocalized with A1 astrocytes. Notch signaling activation contributes to PTX-induced neuropathic pain via A1 astrocytes activation [11].

Caveolin-1 is involved in the regulation of lipoprotein transcytosis, vascular inflammation, and progression of atherosclerosis in the vascular endothelial cell membrane. The increased expression of caveolin-1 regulated the permeability of endothelial cells. Caveolin-1 was significantly upregulated in endothelial cells in mice with high-altitude cerebral edema. Furthermore, hypoxia increased cell permeability, caveolin-1 expression, and claudin-5 internalization and downregulated tight junction proteins in vitro [29, 51]. The expression of caveolin-1 was regulated by HMGB1. HMGB1 expression was increased in the cytoplasm of syncytiotrophoblast cells in preeclampsia placenta. Hypoxic trophoblast-conditioned medium increased endothelial monolayer permeability and increased caveolin-1 protein expression in endothelial cells, which was inhibited by the HMGB1 inhibitor [49, 52]. The HMGB1 inhibitor could also alleviate lipopolysaccharide-induced acute lung injury by upregulating ACE2 and inhibiting the caveolin-1/NF-*κ*B signaling pathway [53]. In this study, rmHMGB1 abolished the protective effect induced by DAPT. However, the inhibitor of caveolin-1, M*β*CD, could protect against the vascular hyperpermeability induced by HMGB1. It might be that the effect of M*β*CD in caveolin inhibition is not specific. However, in several studies, M*β*CD, which removes cellular cholesterol, is still considered as a specific blocker of caveolin-1, and could decrease the expression of caveolins by disrupting caveolar structures [29, 54]. In this study, we only used M*β*CD to suppress the function of caveolin-1 in vitro. We considered that M*β*CD could be qualified for this role in this study. Moreover, chemotherapy drugs may only contact with endothelial cells after administration into the human body because of the limited transport and penetration of chemotherapeutics across the blood–brain barrier. Vascular endothelial cells play an important role in regulating immune responses. Previous studies found that endothelial cells could release inflammatory factors when stimulated [23, 55]. Based on this, immune responses of endothelial cells were performed in this study. All the results showed that the protective effect of DAPT on nab-PTX-induced peripheral neuropathy was related to inhibition of the Notch/HMGB1/caveolin-1 pathway.

Current treatments, which mainly refer to medication, play a limited role in CIPN. It may be because the pathogenesis of CIPN is not fully understood. Targeting inflammatory response might be a new avenue to treat CIPN in animal models. It is important to translate these preclinical findings to clinical applications. In this study, we found inhibition of the Notch pathway by DAPT alleviated CIPN in the rat models. In the current clinical treatment, drugs targeting the Notch pathway mainly focus on Notch ligand monoclonal antibodies, especially DDL3 and DDL4 monoclonal antibodies, which have shown significant effects in the treatment of a variety of tumors. It means that PTX combined with drugs targeting the Notch pathway could increase the efficacy and reduce the side effects of drugs. However, outstanding problems remain in translating findings from animal models to clinical practice. The function and mechanism of the Notch pathway in CIPN of patients are not yet available. It should be elucidated in future studies on the role of CIPN-specific Notch pathways in clinical patients. Based on this, the Notch pathway-related basic research and clinical studies of novel therapeutic approaches should be accelerated. Notch signaling involves in the physiological functions of normal cells, including pluripotent progenitor cell differentiation, apoptosis, cell proliferation, and cell boundary formation. Whether inhibition of Notch signaling affects normal cell function is still unknown. Furthermore, other treatments including nerve blocks, electrical stimulation, intrathecal analgesia pumps, psychological and behavioral interventions also play essential roles in relieving neuropathic pain. The combined application of rehabilitation therapy and drug therapy is effective, which provides a new therapeutic basis for CIPN.

In this study, we mainly focused on the relationship between the activation of spinal Notch signaling pathway and vascular integrality, inflammatory cytokines, and oxidative stress in rat model. However, DAPT is a nonspecific inhibitor of Notch1 receptors, which is a deficiency of this study. Besides, we found that inflammatory cytokines, which are mainly released by microglia and astrocytes in the central nervous system, are increased. Based on this, we hypothesize that microglia may induce the release of inflammatory factors to destroy vascular integrity by activating Notch signaling pathway, but the specific mechanism remains to be explored. In addition, only male rats were used to explore the role of the Notch pathway in neuropathic pain. It is a limitation of this study that female and elderly rats were not conducted. Neuropathic pain is seemingly higher in women than in men and increases significantly with age. The role of the Notch pathway in nab-PTX-induced peripheral neuropathy in female and elderly rats needs to be further studied.

## 5. Conclusion

The findings suggest that activation of the Notch pathway is related to nab-PTX-induced peripheral neuropathy, including mechanical allodynia and thermal hypersensitivity. Inhibition of the Notch pathway by DAPT alleviated nab-PTX-evoked apoptosis, glial response, and microtubule and axonal damage and promoted the restoration of vascular permeability in the rat spinal cord. The protective effect of DAPT was attributed to the decreased levels of proinflammatory cytokines via inhibition of the Notch/HMGB1/caveolin-1 pathway.

## Data Availability

The datasets generated during and/or analyzed during this study are available from the corresponding author on reasonable request.
